# Insight into Genome-Wide Associations of Growth Trajectories Using a Hierarchical Non-Linear Mixed Model

**DOI:** 10.3390/biology15040361

**Published:** 2026-02-20

**Authors:** Ying Zhang, Li’ang Yang, Weiguo Cui, Runqing Yang

**Affiliations:** 1 College of Animal Science and Veterinary Medicine, Heilongjiang Bayi Agricultural University, No. 5 Xinfeng Road, High-Tech Development Zone, Daqing 163319, China; yingzhang@byau.edu.cn (Y.Z.); dqcuiwg@163.com (W.C.); 2 College of Life Science, Northeast Agricultural University, No. 600 Changjiang Road, Xiangfang District, Harbin 150030, China; liangyang@neau.edu.cn; 3 Research Center for Aquatic Biotechnology, Chinese Academy of Fishery Sciences, No. 150 Qingta West Road, Fengtai District, Beijing 100141, China

**Keywords:** growth trajectory, genome-wide association analysis, hierarchical non-linear mixed model, multivariate mixed model, computing efficiency

## Abstract

In applying a hierarchical mixed model to genome-wide association analysis (GWAS) of longitudinal data, dimensionality reduction through modeling repeated measurements improves both computational efficiency and statistical power. Legendre polynomials can flexibly fit population growth trajectories, but higher orders substantially increase computational complexity. Instead of using Legendre polynomials, we first estimated fewer individual-specific parameters using biologically meaningful non-linear models and then associated these phenotypic regressions with genetic markers using a multivariate linear mixed model (mvLMM). After performing a canonical transformation of the regressions based on the pre-estimated covariance matrices under the null genomic mvLMM, we decomposed the mvLMM into mutually independent univariate models and incorporated EMMAX to enable rapid genome-wide mixed-model associations for each transformed phenotype. Simulations for longitudinal association analysis in maize and GWAS for the growth trajectories of body weights in mice demonstrated the advantages of hierarchical non-linear mixed models in computing efficiency and statistical power for detecting quantitative trait loci (QTL), compared with mvLMM for multiple growth points and the hierarchical random regression model using Legendre polynomials as sub-models.

## Simple Summary

Understanding how body weight changes as animals grow helps scientists identify genes that influence health, development, and productivity. Traditional methods analyze body weight at various ages but are slow and often miss important genetic signals. In this study, we developed a faster and more powerful approach that summarizes each animal’s growth pattern using simple biological growth curves instead of many individual measurements. We then linked these growth patterns to genetic markers across the whole genome. Applying this method to a large mouse population, we identified genetic regions that influence how body weight changes over time with much higher computational efficiency and improved detection ability compared with conventional methods. This approach provides a practical way to study genes controlling growth and other time-dependent traits, and can be broadly applied in animal breeding, biomedical research, and studies of development and disease.

## 1. Introduction

During plant and animal development, changes in physiology and performance over time or along quantitative gradients give rise to dynamic or longitudinal traits [1,2,3]. To characterize growth trajectories that vary over time, repeated observations are collected across multiple time points. Such trajectories are influenced by both environmental and genetic factors and have traditionally been analyzed sequentially using univariate or multivariate linear mixed models (mvLMMs) for a limited number of balanced observations, as well as by random regression models (RRMs) [4,5,6]. In contrast, RRMs are generally more appropriate for genetic analyses of dynamic traits because they can flexibly model temporal changes in genetic and environmental effects using many unequally spaced observations.

On a biological basis, dynamic traits are typically regulated by multiple genes whose effects may change, or be switched on or off, over the course of growth [6]. Genetic markers can therefore capture different effects across growth trajectories, as the underlying causal loci may be differentially expressed over time. In linkage analysis for dynamic traits, function mapping [2,7] has been proposed to model genotypic effects using biologically meaningful curves, such as the logistic curve, power function, and the Emax model, while simultaneously accounting for time-dependent residuals with various covariance structures [8]. However, the computing efficiency of these approaches is insufficient for high-throughput genomic data. In particular, non-linear biological models cannot be decomposed into additive polygenic and residual components, making genome-wide application impractical.

With advances in sequencing technologies, RRMs have been increasingly applied in GWAS because they can efficiently identify causal variants that influence the trajectories of dynamic traits. In typical RRM-based association analyses, either polynomial functions of order 2–6 or linear regressions are used to describe changes in marker and polygenic effects over time. However, this requires estimation of covariance matrices of order 3–7, which becomes computationally demanding when testing large numbers of markers, even in moderately sized populations. To alleviate this issue, the EMMAX algorithm [9] has been extended to the RRM framework, and methods such as GMA-fixed and GMA-trans [10,11,12] have further simplified genome-wide longitudinal association analyses.

Theoretically, in longitudinal data analysis, RRMs can be stratified into a hierarchical mixed model when time-dependent effects are characterized through individual-specific parameters of phenotypic trajectories [4,13,14]. This hierarchical mixed model first fits each individual’s phenotypic trajectory and then analyzes the resulting phenotypic parameters using an mvLMM, leading to a substantial reduction in the dimensions of repeated measurements. In hierarchical random regression model (Hi-RRM)-based association analysis [15], we previously fitted individual phenotypic trajectories using Legendre polynomials at the first hierarchy. Although high-order polynomials provided a good global fit for phenotypic trajectories, too many phenotypic regression coefficients analyzed resulted in a dramatic increase in computational burden for association tests at the second hierarchy. In particular, overfitting and Runge’s phenomenon [16] can occur when estimating high-order random regressions.

In this study, instead of Legendre polynomials, we model the individual growth trajectories of body weight in mice using Richards curves (a biologically meaningful non-linear growth function) and then associate the resulting phenotypic regressions with genetic markers using an mvLMM, thereby extending Hi-RRM from linear to non-linear models at the first hierarchy. Furthermore, we apply canonical transformation to the phenotypic regressions [17], which decomposes the mvLMM into multiple independent univariate models and greatly improves the computational efficiency for genome-wide association tests.

## 2. Methods

### 2.1. Modeling Individual Growth Trajectory

Legendre polynomials are widely used to fit the diverse growth trajectories of dynamic traits because of their flexibility and orthogonality. However, high-order terms introduce excessive parameters, inflate the dimensionality of the covariance structures, and reduce computational efficiency in mvLMM association analysis. In contrast, classical biological growth functions, such as Bertalanffy, Gompertz, and Logistic curves, capture the proportional relationship between growth rate and size using only a few parameters and have clear biological interpretations. Extending these, the Richards growth model introduces an additional parameter controlling the inflection point and is expressed as

(1) $$y(t)=a(1+be^{-rt}{)}^{1/\left(1-k\right)},$$

where *y*(*t*) denotes the phenotypic value at age (or time point) *t*; *a* is the asymptotic value representing the mature size; *b* is an integration constant related to the initial condition; *r* is the intrinsic growth rate; and *k* is the shape parameter controlling the position of the inflection point. The Richards model reduces to the Bertalanffy curve when *k* = 0, to the Gompertz curve when *k* = 1, and to the Logistic curve when *k* = 2.

If an individual has enough repeated measurements to fit its growth curve, its phenotypic regression parameters ($a_{i}$, $b_{i}$, $r_{i}$, and $k_{i}$ for the *i*th individual) are estimated via non-linear least squares. Otherwise, best unbiased non-linear prediction [18,19] can be used, regarding phenotypic regressions as random effects. For population-level comparisons among growth models, mean body weights at each age were calculated across individuals. These age-specific means were then fitted using non-linear least squares for biologically meaningful growth curves (Bertalanffy, Gompertz, Logistic, and Richards models) and ordinary least squares for Legendre polynomials of order 0 to 8. Model goodness of fit was evaluated using the Bayesian Information Criterion (BIC), Akaike Information Criterion (AIC), and residual sum of squares.

### 2.2. Genomic Hierarchical Random Regression Model (Hi-RRM)

The genomic Hi-RRM for repeated measurements $y_{i}$ at time points $t_{i}$ on the *i*th individual (i=1, 2, ⋯, n) is specified as

(2) $$\left\{\begin{array}{c} y_{i}=f(p_{i},t_{i})+e_{i}\\ p_{i}=z_{i}a+g_{i}+pe_{i} \end{array}\right.$$

where f(·) represents the Richards growth function in the first hierarchy; $p_{i}=[\begin{array}{cccc} a_{i} & b_{i} & r_{i} & k_{i} \end{array}]$, with $a_{i}$, $b_{i}$, $r_{i}$ and $k_{i}$ being regression coefficients in the Richards model (1) for the *i*th individual, which are subjected to genome-wide association tests using high-throughput genetic markers in the second hierarchy; $e_{i}$ is the vector of the residual errors for the *i*th individual; $z_{i}$ is the genotypic indicator of the tested marker; ***a*** is the vector of genetic effects of the tested marker; $g_{i}$ is a vector of the polygenic effects; and $pe_{i}$ is a vector of the permanent environmental effects on phenotypic regressions for the *i*th individual.

We assume that $g_{i}\sim N(0,\;V_{g})$, with covariance matrix $V_{g}$ for polygenic regression effects; $pe_{i}\sim N(0,\;V_{pe})$, with covariance matrix $V_{pe}$ for permanent environmental effects; and $e_{i}\sim N(0,\;I_{4}\sigma_{e}^{2})$, where $I_{4}$ is a 4 × 4 identity matrix and $\sigma_{e}^{2}$ is the residual variance.

### 2.3. Statistical Inference

#### 2.3.1. mvLMM for Regression Phenotypes

At the second hierarchy, the regression parameters estimated from the individual growth curves are treated as a set of correlated quantitative traits. The objective of the second hierarchy is to associate these multivariate phenotypes with genetic markers while accounting for both their genetic covariance and the genomic relationship among individuals.

The covariance matrices $V_{g}$ and $V_{pe}$ are estimated under the null genomic mvLMM with multivariate GEMMA [20]. Statistical significance of variance and covariance components was assessed using Wald-type tests based on the ratio of estimates to their standard deviations. Based on both covariance matrices, we canonically transform genomic mvLMM to multiple independent univariate models [21,22], and we associate multiple phenotypic regressions with genetic markers using the mvRunKing method [15].

For the sake of stating association tests, we rewrite mvLMM at the second hierarchy in the notation of matrix:

(3) P=z⊗a+G+E ,

with $P={\left[\begin{array}{cccc} p_{1} & p_{2} & \ldots & p_{n} \end{array}\right]}^{T}$, $G=[\begin{array}{cccc} g_{1} & g_{2} & \ldots & g_{n} \end{array}{]}^{T}$, and $E=[\begin{array}{cccc} pe_{1} & pe_{2} & \ldots & pe_{n} \end{array}{]}^{T}$.

Here, **G** and **E** follow matrix normal distributions [20], denoted by $G\sim \mathrm{MN}(0,\;V_{g},\;K)$ and $E\sim \mathrm{MN}(0,\;V_{pe},\;I_{n})$ with genomic relationship matrix K [23,24].

A transformation matrix $L=U_{\lambda }^{T}(S_{pe}^{-\frac{1}{2}}U_{pe}^{T})$ is calculated by eigen-decomposing $S_{pe}=U_{pe}V_{pe}U_{pe}^{T}$ and $(S_{pe}^{-\frac{1}{2}}U_{pe}^{T})V_{g}(S_{pe}^{-\frac{1}{2}}U_{pe}^{T}{)}^{T}=U_{\lambda }S_{\lambda }U_{\lambda }^{T}$, where $S_{pe}$ and $S_{\lambda }$ are diagonal matrices of eigenvalues, and $U_{pe}$ and $U_{\lambda }$ are eigenvector matrices corresponding to $V_{pe}$ and $(U_{pe}S_{pe}^{-\frac{1}{2}}U_{pe}^{T})V_{g}(U_{pe}S_{pe}^{-\frac{1}{2}}U_{pe}^{T}{)}^{T}$, respectively. Let $P^{*}=PL^{T}$, $a^{*}=aL^{T}$, $G^{*}=GL^{T}$, and $E^{*}=EL^{T}$, and we canonically transform model (3) to

(4) $$P^{*}=z⊗a^{*}+G^{*}+E^{*}\;,$$

According to $V(P^{*})=V(G^{*}+E^{*})=S_{\lambda }⊗K+I_{4n}$, model (4) is divided into mutually independent

(5) $$p_{l}^{*}=za_{l}^{*}+g_{l}^{*}+e_{l}^{*}\;,$$

for l=1, 2, 3, 4.

Here, $p_{l}^{*}$, $a_{l}^{*}$, $g_{l}^{*}$, and $e_{l}^{*}$ are the *l*th columns of $P^{*}$, $a^{*}$, $G^{*}$, and $E^{*}\;$, respectively. $V(p_{l}^{*})=s_{\lambda }^{l}K+I_{n}$, is obtained, with $s_{\lambda }^{l}$ denoting the *l*th element of $S_{\lambda }$.

#### 2.3.2. EMMAX-Based Association Analysis

Next, we implement association tests in EMMAX for each transformed phenotype. By spectrally decomposing $K=U_{k}S_{k}U_{k}^{T}$, with $S_{k}$ and $U_{k}$ being the corresponding eigenvalue and eigenvector matrices, $V^{-1}(p_{l}^{*})$ is factorized into $V^{-0.5}\cdot V^{-0.5}$, where $V^{-0.5}=(s_{\lambda }^{l}S_{k}+I_{n}{)}^{\frac{1}{2}}U_{k}^{T}$. Let $p_{l}^{\prime }=V^{-0.5}p_{l}^{*}$, $z^{\prime }=V^{-0.5}z$, and $e_{l}^{\prime }=V^{-0.5}e_{l}^{*}$; we eliminate the dependence among individuals [25,26,27], transforming model (5) to a simple linear regression model:

(6) $$p_{l}^{\prime }=z^{\prime }a_{l}^{*}+e_{l}^{\prime }.$$

Using the least squares, we infer the marker effect with the statistic

(7) $$\chi_{l}^{2}=({\hat{a}}_{l}^{*}{)}^{2}[V\left({\hat{a}}_{l}^{*}\right){]}^{-1},$$

where ${\hat{a}}_{l}^{*}$ and $V({\hat{a}}_{l}^{*})$ are the estimated genetic effect and its variance for the tested marker.

From four independent models (6), we sum up the statistics (7) obtained to test the pleiotropic QTL by

(8) $$\chi^{2}=\sum_{l=1}^{4}\chi_{l}^{2},$$

which follows a Chi-squared distribution of d degrees-of-freedom.

Within the EMMAX framework, the polygenic variance for tested markers is approximated by the genomic variance $s_{\lambda }^{l}$ (or equivalently, heritability $\frac{s_{\lambda }^{l}}{1+s_{\lambda }^{l}}$). This approximation may increase false negative errors. To improve the statistical power for detecting QTLs, we re-estimate the polygenic heritability starting from the genomic heritability, focusing on markers with higher significance levels or larger effects for each model (6) [28]. This optimization process will run for at most two rounds of EMMAX.

### 2.4. Data Description

#### 2.4.1. Simulated Phenotype

A total of 300,000 single-nucleotide polymorphisms (SNPs) genotyped on 2640 maize [29] were used to simulate longitudinal association analysis. Without loss of generality, we described phenotypic trajectory using the Richards growth model. A total of 500 QTLs were randomly distributed over all genomic SNPs. Population regression coefficients and residual variances were assigned in the same way as in real data analysis. As simulated in Hi-RRM [15], we generated longitudinal phenotypic values at the 16 growth points by setting all correlations between additive genetic or permanent environmental regressions to 0.5, and permanent environmental regression variances to 4.0, at a regression heritability of 0.5. SNPs were designated as QTLs if they had the highest test statistic among the 20 closest neighboring SNPs. Statistical powers were defined as the percentage of the identified QTLs over the total number of simulated QTLs. Under good genomic control, the receiver operating characteristic ROC curves were plotted to show the statistical power for detecting QTLs against a range of Type I error rates. To investigate the statistical performance of the hierarchical non-linear mixed model, we repeated the simulation 50 times, each with varying positions and effects of simulated QTLs, and recorded the average results.

#### 2.4.2. Real Phenotype

To investigate rapid and extreme size evolution in island mice, a large F2 intercross between Gough Island and WSB/EiJ mice was constructed to identify QTLs underlying variation in the evolution of body weight trajectories [30]. Body weights were collected weekly from 1 to 16 weeks of age for a total of 1374 F2 mice. At 16 weeks of age, the mice were euthanized by CO_2_ asphyxiation or by decapitation. Liver samples were collected and stored at −80 °C. For genetic analysis, the samples were sent to GeneSeek (Neogen, Lincoln, NE, USA) for DNA extraction. The approximately 77.8 K Mega Mouse Universal Genotyping Array (MegaMUGA, The Jackson Laboratory, Bar Harbor, ME, USA) was used to genotype the DNA samples. After strict quality control, a total of 11,833 SNPs from 1212 mice were retained for GWAS of growth trajectories.

## 3. Results

### 3.1. Simulation Analysis

**Figure 1:**
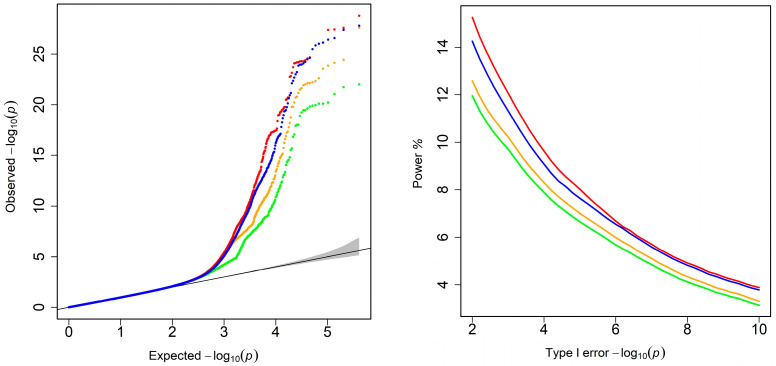
Q-Q profiles (**left**) and ROC profiles (**right**) for the simulated phenotypes. The red, green, orange and blue represent the Richards model and Legendre polynomials of 3, 4, and 6 orders, respectively.

We fitted the simulated longitudinal phenotypes using the Richards model and Legendre polynomials of 3, 4 and 6 orders, and associated the resulting phenotypic regressions with markers using the mvLMM. In the first step, the fourth-order polynomial provides goodness of fit comparable to the Richards model, while the sixth-order polynomial emerged as the optimal fitting model despite exhibiting signs of overfitting, with a residual variance lower than the target value of 4.0. Figure 1 displays the Q-Q and ROC plots for four fitting models in the second step. The Q-Q plots illustrate that the statistical *p*-values fit well with the expected distribution; severe inflation was observed for a small number of markers with high *p*-values, and genomic control values were all close to 1. In terms of statistical power to detect QTLs, the Richards model was consistently superior to polynomials, and such differences between the Richards model and polynomials decreased with the increased orders of the Legendre polynomials, so that the optimal sixth-order polynomial was very close to the Richards model at more than the Bonferroni correction significance level, which suggested the necessity of choosing a biologically meaningful growth model with fewer estimated parameters.

### 3.2. Real Data Analysis

#### 3.2.1. Phenotypic Variation and Population-Level Fitting of Growth Curves

**Figure 2:**
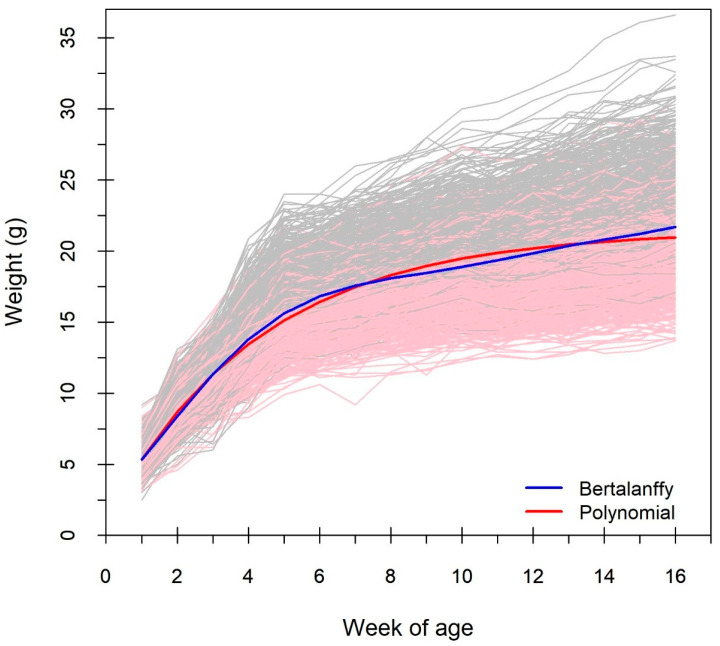
Plots of body weights to weeks of age in mice. The gray and pink represent male and female growth curves, respectively.

In Figure 2, body weights from 1 to 16 weeks of age were plotted for 1212 mice. The longitudinal profiles revealed increasing phenotypic variability with age, an early inflection point in growth, and substantial heterogeneity in growth trajectories across individuals, particularly between the sexes. To characterize the population-level growth pattern, we fitted the biological meaningful non-linear growth curves (Bertalanffy, Gompertz, Logistic, and Richards) and Legendre polynomials of order 0–8 to the mean body weights across ages. According to the goodness-of-fit statistics presented in Table 1 and Table S1, the Bertalanffy curve and sixth-order Legendre polynomial, which showed the lowest BIC values, were selected as the optimal non-linear (biologically meaningful) and linear models, respectively, as follows:

$$y=21.428(1-0.948e^{0.234t}),$$

$$y=16.946+6.564\psi_{1}(t)-3.225\psi_{2}(t)+1.976\psi_{3}(t)-0.518\psi_{4}(t)-0.352\psi_{5}(t)+0.299\psi_{6}(t),$$

with $\psi_{i}(t)$ (i=1, 2, ⋯, 6) being described in [31].

Although the Legendre polynomial achieved a better overall fit, the Bertalanffy curve closely overlapped with it at early (1st–3rd weeks) and specific later (7th and 13th weeks) developmental stages (Figure 1) and required fewer parameters.

#### 3.2.2. Estimation of Regression Parameters and Covariance Structures

The genomic and permanent environmental covariance matrices of regression parameters for the Bertalanffy curves, ${\hat{V}}_{g}$ and ${\hat{V}}_{pe}$, were estimated under the null second hierarchical model (i.e., with no QTLs), denoted by

$$\left[\begin{array}{ccc} 0.091\;(0.024) & 0.020\;(0.021) & -0.043\;(0.022)\;\\ 0.020\;(0.021) & 0.126\;(0.036) & 0.075\;(0.029)\\ -0.043\;(0.022)\; & 0.075\;(0.029) & 0.102\;(0.032) \end{array}\right]$$

and

$$\left[\begin{array}{ccc} 0.640\;(0.027) & -0.131\;(0.022) & -0.441\;(0.026)\\ -0.131\;(0.022) & 0.776\;(0.033) & 0.569\;(0.030)\\ -0.441\;(0.026) & 0.569\;(0.030) & 0.879\;(0.037) \end{array}\right]$$

Values in parentheses indicate standard deviations.

Most variance and covariance components were significantly different from zero (*p* < 0.01), except for the genomic covariances between parameters a and b, and between a and r. The corresponding ${\hat{V}}_{g}$ and ${\hat{V}}_{pe}$ for the sixth-order Legendre polynomial are presented in Table S1. These results supported the feasibility of modeling genetic covariance among growth-curve parameters.

#### 3.2.3. Genome-Wide Detection of QTLs Using Hi-RRM

**Figure 3:**
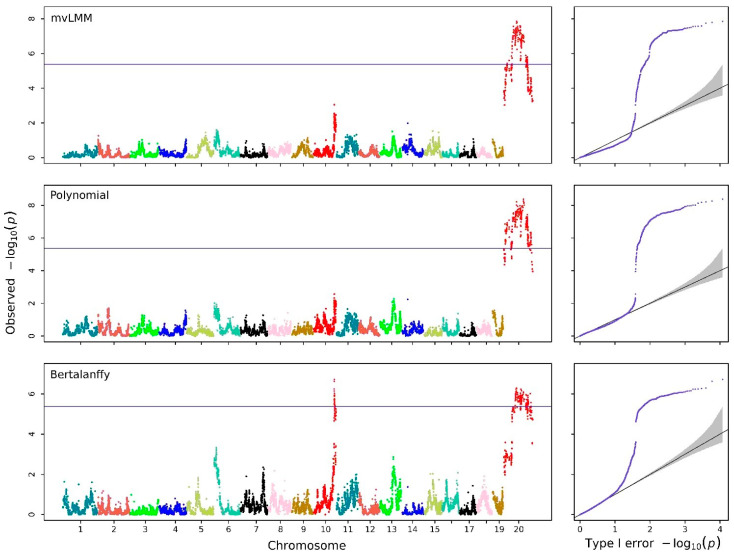
Manhattan and Q-Q plots obtained with mvLMM for 16 growth points and the Hi-RRMs for the Legendre polynomial and Bertalanffy growth curve of body weights in mice. The horizontal reference lines in the Manhattan plots represent Bonferroni correction thresholds at a significance level of 5%.

Genome-wide association analyses were performed using Hi-RRM based on the Bertalanffy curve and the sixth-order Legendre polynomial, with mvLMM applied to body weights at 16 individual time points as a comparison (Figure 3). By jointly testing the regression coefficients within the hierarchical random regression framework, each SNP was evaluated for its overall association with the entire growth trajectory. Q-Q plots indicated that both Hi-RRM approaches produced well-calibrated test statistics with limited inflation, whereas mvLMM showed substantial deviation from the expected distribution, consistent with underfitting. The genomic control values were 3.321 for the Bertalanffy-based Hi-RRM, 7.683 for the Legendre-based Hi-RRM, and 14.981 for mvLMM. The Bertalanffy-based Hi-RRM detected one additional QTL relative to the other two methods.

In the Manhattan plot obtained from the Bertalanffy-based Hi-RRM, 267 SNPs had −log10(p) values exceeding the Bonferroni-adjusted significance threshold (5.374) at a significance level of 0.05. Among the top six significant SNPs (see Table 2 for details), three independent SNPs were identified as QTLs associated with the growth trajectory of body weight in mice. These QTLs were located at UNC18848064 on chromosome 10, JAX00714218, and UNC31155388 on chromosome 20.

#### 3.2.4. Associations with Biologically Interpretable Growth Parameters

**Figure 4:**
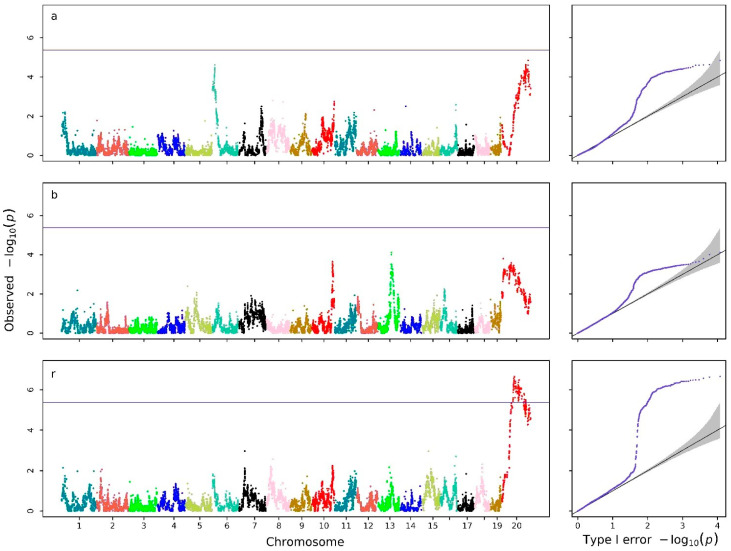
Manhattan and Q-Q plots obtained with the Hi-RRMs for a, b, and r in the Bertalanffy curve of body weights in mice. The horizontal reference lines in the Manhattan plots represent Bonferroni correction thresholds at a significance level of 5%.

For the Bertalanffy growth model, the regression coefficients had biological meanings: a represents the limit growth, b the initial growth, and r the intrinsic growth rate. To further interpret the biological basis of the QTLs detected from the joint trajectory-level analysis, we conducted separate genome-wide mixed-model association analysis for each Bertalanffy parameter (a, b, and r) and plotted their corresponding Manhattan and Q-Q plots in Figure 4. With well-controlled false positive statistical errors (genomic control values of 1.068, 1.021, and 1.088), two QTLs overlapping with QTLs were detected on chromosome 20 for the intrinsic growth rate, while two distinct signals were observed on chromosomes 6 and 20, respectively, which might be associated with the limit growths.

#### 3.2.5. Time-Dependent Genetic Effects of Detected QTLs

As described in model (2), the phenotypic regressions were assumed to be linear and additive at the second hierarchy, but genetic regression effects were non-linear and non-additive in the individuals’ Richards curves at the first hierarchy. This suggested that the estimated genetic regression effects could not be directly substituted into the Bertalanffy curve to calculate genetic effects on body weights. Nevertheless, the genetic effect of the ith QTL on body weights should be evaluated as the difference between the Bertalanffy curve of the population and that associated with the i-th QTL, as follows:

$$g_{i}(t)=(21.428+a_{i})(1-(0.948+b_{i})e^{-(0.234+r_{i})t})-21.428(1-0.948e^{-0.234t})$$

**Figure 5:**
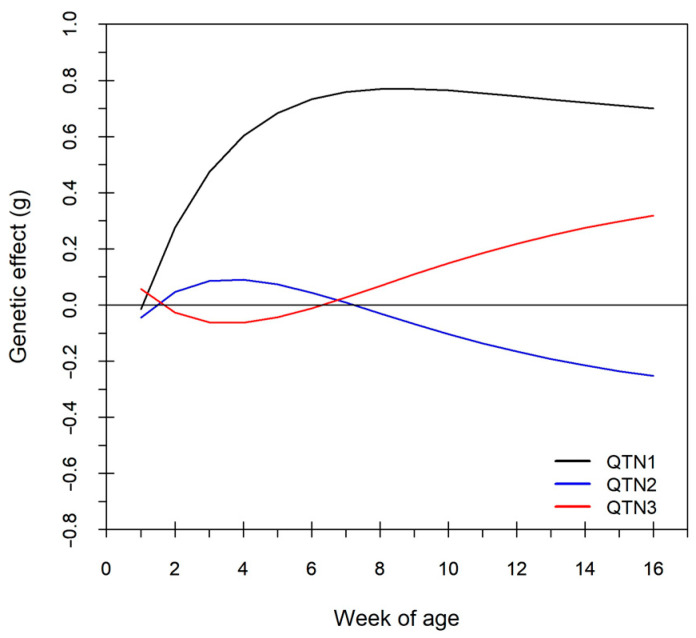
Changes in genetic effects of the 3 detected SNPs on body weights in mice across age.

Changes in the genetic effects of the three detected QTLs were plotted across the measuring period (Figure 5). Among the three plots, QTL1 showed a positive effect on the growth trajectory, following an approximately parabolic pattern from 1 to 16 weeks of age. QTL2 exhibited a spoon-shaped pattern where the genetic effects decreased rapidly before the four th week of age and then increased linearly afterward, while the change in genetic effects of QTL3 was almost symmetrical to that of QTL2. Moreover, the three remaining significant SNPs showed similar genetic effect changes to those of their highly linked QTLs (Figure 5).

## 4. Discussion

Under the assumption that the parameters of the growth curve were linear and additive at the second hierarchy, the Hi-RRM could fit individuals’ growth trajectories by using either Legendre polynomials or biologically meaningful non-linear models at the first hierarchy [14,32]. Because non-linear growth curves required fewer estimated phenotypic parameters (regression coefficients) than those obtained with Legendre polynomials, the Hi-RRM achieved higher computational efficiency and greater statistical power for detecting QTLs. In the estimation of variance components or association tests, an excess number of phenotypic regressions might lead to non-convergence of the mvLMM, especially for small resource populations with limited sample sizes relative to the number of parameters. The QTLs for parameters in the non-linear growth curve, even though they overlapped with the detected QTLs, were further identified to reveal genome-wide association with the features of growth trajectory. Unlike our proposed Hi-RRM, which used mv-LMM in the second step to map QTLs for growth trajectories, these longitudinal association analyses [33,34,35] mainly employ LM or LMM to separately infer the significance of individual regression coefficients or specified growth points. This significantly reduced the statistical power to detect QTLs.

Although Legendre polynomials provided a better fit to the population growth trajectory than the Bertalanffy curve, the Hi-RRM based on Legendre polynomials identified no more QTLs than that based on the Bertalanffy curve. To optimally fit individual growth trajectories at the first hierarchy, researchers typically estimated phenotypic parameters by using non-linear mixed models with different variance functions for the residuals [19]. However, this approach substantially increased computational complexity and led to a decrease in the precision of parameter estimates. In practice, we also adopted the RRM, taking the Bertalanffy curve as a sub-model and considering five different variance functions for the residuals (see Table S2) at the first hierarchy. Using the mvLMM for the estimated phenotypic parameters, we identified only a single QTL on chromosome 10 (see Figure S1). Therefore, unless the longitudinal data are unbalanced, non-linear mixed models are not recommended for estimating phenotypic regression coefficients. This suggests that, at the first hierarchy, individual phenotypic regressions in either linear or non-linear growth curves could be efficiently estimated using linear or non-linear least squares methods when longitudinal data are balanced or sufficiently dense to model each individual’s phenotypic trajectory.

In terms of computational efficiency, most of the computing time in Hi-RRM was spent estimating the regression covariance matrices under the null model and conducting association tests. When linear and non-linear growth curves involved the same number of parameters, both the Hi-RRM for Legendre polynomials [15] and the Hi-RRM based on the non-linear growth curve proposed in this paper estimated the regression covariance matrices with a computational complexity of O(*n*^2^*d*^2^*m*). However, the Hi-RRM required substantially less computational time for association tests with non-linear growth curves O(*dn*^2^*m*) than with Legendre polynomials O(*dn*^2^*m*), particularly given the relatively small number of parameters (*d*). Data analysis demonstrated that the Hi-RRM based on the Bertalanffy curve completed the association tests in under one minute, whereas the Hi-RRM [15] required about 30 min for the same task.

## 5. Conclusions

Using the Bertalanffy curve to characterize the growth trajectory of body weights in mice, the hierarchical non-linear mixed model identified one additional QTL on chromosome 10, alongside those on chromosome 20, with nearly 100-fold higher computational efficiency than the hierarchical linear model using sixth-order Legendre polynomials as sub-models. This demonstrated the advantages of the hierarchical non-linear mixed model with respect to both computational efficiency and statistical power for detecting quantitative trait loci, compared with the mvLMM for multiple time points and the hierarchical random regression model.

## Figures and Tables

**Table 1:** Goodness of fit to 5 growth curves in mouse.

Model	Bertalanffy	Gompertz	Logistic	Richards	Legendre Polynomial
No. of parameters	3	3	3	4	7
BIC	30.597	38.731	45.661	31.375	19.058
AIC	27.507	35.641	42.571	27.512	12.877
R^2^	0.991	0.985	0.976	0.992	0.998
Residual variance	0.211	0.351	0.542	0.187	0.086

**Table 2:** The QTLs detected with Hi-RRM for Bertalanffy growth curves in mouse.

QTL	Chr	SNP	Pos.	MAF	a	b	r	−log10(p)
1	10	UNC18848064	119665663	0.486	0.638 (0.240)	0.021 (0.006)	0.012 (0.005)	6.725
	10	UNC18846270	119558507	0.490	0.571 (0.241)	0.021 (0.006)	0.013 (0.005)	6.631
	10	UNC18844677	119461165	0.490	0.556 (0.241)	0.021 (0.006)	0.013 (0.005)	6.246
2	20	JAX00714218	75031133	0.494	−0.342 (0.115)	0.009 (0.003)	0.012 (0.002)	6.293
	20	JAX00180944	75809493	0.491	−0.338 (0.116)	0.009 (0.003)	0.012 (0.002)	6.269
3	20	UNC31155388	99388659	0.483	0.430 (0.117)	−0.009 (0.003)	−0.012 (0.002)	6.241

## Data Availability

The data used in this research were downloaded from https://phenome.jax.org/projects/Payseur1 (accessed on 3 September 2024).

## Supplementary Materials

The following supporting information can be downloaded at https://www.mdpi.com/article/10.3390/biology15040361/s1, Figure S1: Manhattan and Q-Q plots obtained with d the Hi-RRMs for Legendre polynomial and Bertalanffy growth curves with constant residual variance function. Table S1: Population-level goodness of fit for Legendre polynomials (orders 0–8) and corresponding regression covariance estimates under the null hierarchical model. Table S2: Goodness of fit for 5 residual variance functions obtained with the Hi-RRM for Bertalanffy curves.

## Abbreviations

The following abbreviations are used in this manuscript:

mvLMMs	Multivariate linear mixed models
RRMs	Random regression models
Hi-RRM	Hierarchical random regression model
BIC	Bayesian Information Criterion
AIC	Akaike Information Criterion
