# Supplementary material for: Insight into Genome-Wide Associations of Growth Trajectories Using a Hierarchical Non-Linear Mixed Model
Source: Biology (Basel). 2026 Feb 20;15(4):361. doi: 10.3390/biology15040361 (PMC12937830; doi:10.3390/biology15040361)
Supplement: Supplementary file 1 [file biology-15-00361-s001.zip › biology-4094894-supplementary.pdf]

Table S1 Population-level goodness-of-fit for Legendre polynomials (orders 0–8) and corresponding regression covariance estimates under the null hierarchical model.

**Order**	**0**	**1**	**2**	**3**	**4**	**5**	**6**	**7**	**8**
BIC	99.887	73.776	52.504	22.636	20.735	19.390	19.058	21.826	20.843
AIC	98.342	71.458	49.414	18.773	16.100	13.982	12.877	14.873	13.117
R^2	0.929	0.988	0.997	0.999	0.999	0.999	0.999	0.999	0.999
Residual variance	22.715	3.735	0.831	0.108	0.081	0.062	0.051	0.051	0.041

$$\hat{\boldsymbol{V}}_g = \begin{bmatrix} 0.601(0.139) & 0.307(0.084) & -0.203(0.054) & 0.135(0.042) & -0.047(0.024) & -0.061(0.023) & 0.081(0.028) \\ 0.307(0.084) & 0.258(0.067) & -0.081(0.033) & 0.025(0.026) & -0.009(0.016) & -0.039(0.016) & 0.026(0.018) \\ -0.204(0.054) & -0.081(0.033) & 0.113(0.029) & -0.082(0.021) & 0.021(0.011) & 0.035(0.011) & -0.049(0.014) \\ 0.135(0.042) & 0.025(0.026) & -0.082(0.021) & 0.068(0.019) & -0.018(0.009) & -0.021(0.009) & 0.036(0.011) \\ -0.047(0.024) & -0.009(0.016) & 0.021(0.011) & -0.018(0.009) & 0.015(0.007) & 0.001(0.005) & -0.015(0.007) \\ -0.061(0.023) & -0.039(0.016) & 0.035(0.011) & -0.021(0.009) & 0.001(0.005) & 0.018(0.006) & -0.015(0.006) \\ 0.081(0.028) & 0.026(0.018) & -0.049(0.014) & 0.036(0.011) & -0.015(0.007) & -0.015(0.006) & 0.027(0.009) \end{bmatrix}$$

$$\hat{\boldsymbol{V}}_{pe} = \begin{bmatrix} 2.726(0.116) & 1.509(0.084) & -0.544(0.043) & 0.154(0.034) & -0.082(0.032) & -0.053(0.025) & 0.079(0.028) \\ 1.509(0.084) & 1.686(0.071) & -0.199(0.032) & -0.186(0.027) & 0.068(0.025) & -0.088(0.020) & 0.025(0.022) \\ -0.544(0.043) & -0.199(0.032) & 0.647(0.028) & -0.076(0.017) & -0.148(0.016) & 0.072(0.013) & -0.110(0.014) \\ 0.154(0.034) & -0.186(0.027) & -0.076(0.017) & 0.455(0.019) & -0.097(0.013) & -0.174(0.012) & 0.123(0.012) \\ -0.082(0.032) & 0.068(0.025) & -0.148(0.016) & -0.097(0.013) & 0.407(0.017) & 0.014(0.010) & -0.200(0.012) \\ -0.053(0.025) & -0.088(0.020) & 0.072(0.013) & -0.174(0.012) & 0.014(0.010) & 0.261(0.011) & -0.034(0.009) \\ 0.079(0.028) & 0.025(0.022) & -0.110(0.014) & 0.123(0.012) & -0.200(0.012) & -0.034(0.009) & 0.313(0.013) \end{bmatrix}$$

Table S2 Goodness of fit for 5 residual variance functions obtained with the Hi-RRM for Bertalanffy curves

**Residual Variance function**	**Function Expression**	**BIC**	**AIC**
Homogenous variance	0.6613	57098.96	56980.87
Constant variance function*	$5.8352 \times 10^{-8}\rho$	55073.25	54837.07
Power variance function	$t^{-0.2580}$	57874.46	57748.50
Constant plus power variance function	$(7.0310\times10^{-11} + t^{-0.1356})^2$	57927.38	57927.38
Exponential variance function	$e^{-0.0569t}$	57797.77	57671.81

$\rho = (1.00, 2704.13, 3808.78, 4546.61, 7112.35, 4047.86, 2990.93, 2320.94, 2638.16, 2498.68, 3091.68, 2680.94, 2386.29, 2929.82, 2909.22, 3998.56)$

*Optimal residual variance function.

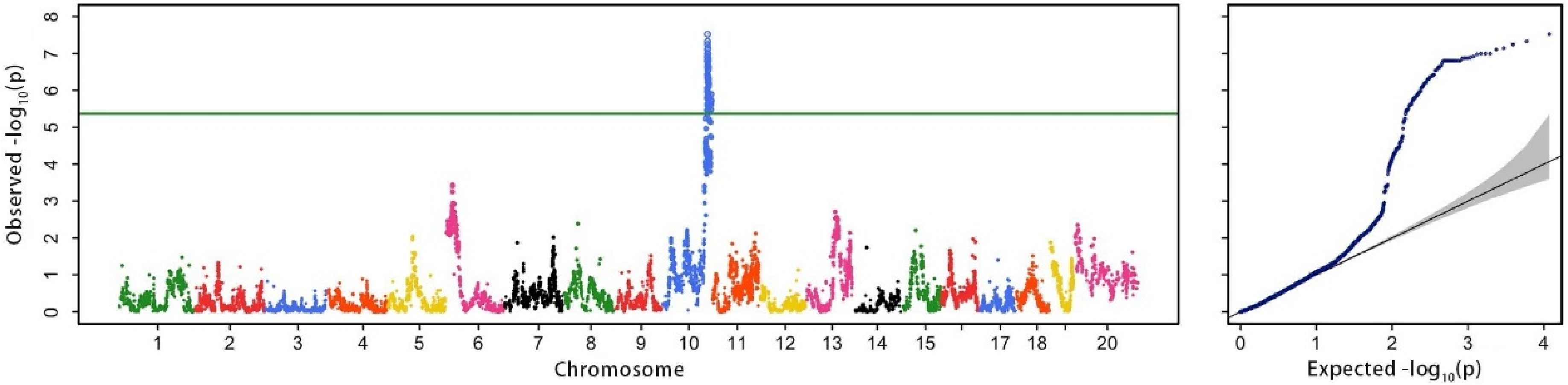

Figure S1 Manhattan and Q-Q plots obtained with d the Hi-RRMs for Legendre polynomial and Bertalanffy growth curves with constant residual variance function. The horizontal reference lines in Manhattan plots represent Bonferroni correction thresholds at a significance level of 5%.
